# Predictive Value of the Surgical Apgar Score for Major Postoperative Complications: A Prospective Observational Study in a Tertiary Care Setting

**DOI:** 10.7759/cureus.101962

**Published:** 2026-01-21

**Authors:** Kamala Kannan Murugan, Emil Phinehas Mariantony, Sasikala Kathiresan

**Affiliations:** 1 General Surgery, Velammal Medical College Hospital and Research Institute, Madurai, IND; 2 General Surgery, All India Institute of Medical Sciences, Madurai, Madurai, IND; 3 Obstetrics and Gynaecology, All India Institute of Medical Sciences, Madurai, Madurai, IND

**Keywords:** intraoperative tool, positive predictive value, postoperative complications, risk prediction, surgical apgar score

## Abstract

Background: The Surgical Apgar Score (SAS) is a simple intraoperative scoring system proposed to estimate postoperative risk; however, its discriminative performance in heterogeneous surgical populations remains uncertain. This study prospectively evaluated the discriminative ability of the SAS for postoperative complications in a mixed surgical cohort.

Methods: In this prospective observational cohort study, adult patients (≥18 years) undergoing elective or emergency major surgical procedures, defined as operations performed under general or regional anesthesia with an anticipated postoperative hospital stay exceeding 48 hours, in general surgery and orthopaedics at a tertiary care center, were enrolled. Intraoperative variables were prospectively collected, and the SAS was calculated for each case. The primary outcome was the occurrence of clinically relevant postoperative complications within 30 days, excluding blood transfusion. Associations were assessed using multivariable logistic regression. Discrimination was evaluated using receiver operating characteristic (ROC) analysis, with prespecified sensitivity analysis including American Society of Anesthesiologists (ASA) physical status.

Results: A total of 95 patients were included (mean age 52.5 ± 18.7 years; 48.4% male). Clinically relevant postoperative complications occurred in 40.4% of patients. Lower SAS was associated with higher complication rates on unadjusted analysis. In the primary multivariable model adjusted for age, duration of surgery, and urgency of procedure, the SAS was not independently associated with the outcome. In sensitivity analysis including ASA physical status, the association between the Surgical Apgar Score and postoperative complications reached statistical significance (p = 0.035) and should be interpreted cautiously. ROC analysis demonstrated limited to modest discriminative ability (area under the curve (AUC) 0.623, 95% CI 0.517-0.728).

Conclusion: In this prospective heterogeneous surgical cohort, the SAS demonstrated limited to modest discriminative ability for clinically relevant postoperative complications. The findings suggest limited utility of the SAS as a standalone risk stratification tool in heterogeneous surgical populations. At most, the score may provide complementary intraoperative risk information when interpreted alongside established preoperative risk measures and clinical judgment. Further validation in larger, procedure-specific cohorts with standardized outcome definitions and internal validation is required.

## Introduction

Postoperative complications remain a major source of morbidity, mortality, and healthcare utilization among surgical patients. Reported rates of postoperative complications within 30 days following major surgery vary widely, ranging from approximately 5% to over 40%, depending on patient characteristics, procedural complexity, and definitions used [1]. A substantial proportion of patients who develop postoperative morbidity subsequently experience prolonged hospital stays, unplanned intensive care unit (ICU) admissions, or mortality, highlighting the importance of effective perioperative risk assessment strategies [2]. Accurate prediction of postoperative risk is therefore essential to optimize patient outcomes, allocate resources efficiently, and guide perioperative decision-making [3].

Several tools are currently used for perioperative risk stratification. The American Society of Anesthesiologists (ASA) physical status classification remains one of the most widely applied systems; however, it is limited by subjectivity and interobserver variability [4,5]. More comprehensive prediction models, such as the American College of Surgeons National Surgical Quality Improvement Program (ACS NSQIP) Surgical Risk Calculator, demonstrate strong predictive performance in large datasets but may show reduced accuracy when applied to smaller or institution-specific populations [4-6]. Importantly, many commonly used risk assessment tools rely predominantly on preoperative variables and do not incorporate intraoperative physiological data, despite the recognized influence of intraoperative events on postoperative outcomes [7].

To address this gap, Gawande et al. introduced the Surgical Apgar Score (SAS) in 2007, a simple 10-point intraoperative scoring system based on estimated blood loss (EBL), lowest heart rate (HR), and lowest mean arterial pressure (MAP) [8]. The SAS was designed to provide an objective, immediately available assessment of intraoperative risk. Since its introduction, numerous studies have evaluated its association with postoperative morbidity and mortality across a range of surgical specialties. While several retrospective and procedure-specific studies have reported favorable predictive performance of the SAS, particularly in high-risk or homogeneous surgical populations, reported discrimination has varied substantially across settings [9].

A recent systematic review synthesizing evidence from multiple surgical disciplines demonstrated that the predictive accuracy of the SAS is context-dependent, with performance influenced by surgical specialty, patient selection, and outcome definitions [10]. Moreover, many validation studies have been retrospective in design, and findings from prospective evaluations, especially in mixed or heterogeneous surgical cohorts, have been inconsistent. These limitations underscore the need for further prospective studies assessing the performance of the SAS in real-world clinical settings that include both elective and emergency procedures.

The present study was therefore designed to prospectively evaluate the predictive performance of the SAS for major postoperative complications in a heterogeneous cohort of adults undergoing major general surgical and orthopaedic procedures at a tertiary care center. In addition, we aimed to examine the performance of the SAS in relation to established clinical risk factors and to explore its potential role as part of a multifactorial perioperative risk assessment strategy.

## Materials and methods

Study design and setting

This was a prospective observational cohort study conducted at the Pondicherry Institute of Medical Sciences (PIMS), Ganapathichettikulam, Puducherry, India, between October 2022 and May 2024. The study was approved by the Institutional Ethics Committee of PIMS (Approval No: IEC:RC/2022/70) and conducted in accordance with the Declaration of Helsinki. Written informed consent was obtained from all participants prior to enrollment.

Participants

All adult patients (≥18 years) undergoing elective or emergency surgery in the departments of General Surgery or Orthopaedics during the study period were eligible for inclusion. Patients were excluded if they declined informed consent, had incomplete intraoperative records required for SAS calculation, underwent minor or day-care procedures, or were lost to follow-up within 30 days postoperatively. The cohort intentionally represented a heterogeneous surgical case-mix, including elective and emergency procedures from general surgery and orthopaedics, to evaluate the performance of the SAS in a real-world mixed surgical population. For the purpose of this study, major surgery was operationally defined as any operative procedure performed under general or regional anesthesia, involving incision beyond superficial soft tissue, and anticipated postoperative hospital stay exceeding 48 hours, including both elective and emergency procedures.

Data collection and variables

Clinical and perioperative data were prospectively collected from electronic medical records using a standardized data extraction form. Recorded variables included: Age (years), sex, surgical department (General Surgery or Orthopaedics), type of surgery (elective or emergency), ASA physical status classification (I-VI), body weight (kg), duration of surgery (minutes), presence of medical comorbidities (yes/no) and previous surgical history (yes/no). Detailed procedure-level classifications (e.g., specific operative procedures, surgical approach, or wound contamination class) were not recorded a priori as the study was designed to evaluate the overall performance of the SAS in a real-world heterogeneous surgicalpopulation. Consequently, procedure-specific analyses were not performed.

Surgical Apgar Score

The SAS was calculated intraoperatively based on EBL, lowest HR, and lowest MAP, as originally described by Gawande et al. [6] EBL was assessed by the operating anesthesia and surgical teams using standard institutional practice, incorporating suction canister volume after subtraction of irrigation fluids and visual assessment of blood-soaked surgical sponges; formal gravimetric sponge weighing was not routinely performed.

HR and MAP were continuously monitored using standard intraoperative anesthetic monitors. The lowest recorded HR and MAP during the operative period were obtained from the anesthesia record. Transient monitor artifacts or non-physiologic readings (e.g., related to electrocautery interference or brief signal loss) were excluded based on clinical judgment by the anesthesia team, and sustained values were considered for SAS calculation. Measurements were taken over the full duration of surgery rather than a predefined time window, consistent with the original SAS methodology.

Outcome definition

The primary outcome was the occurrence of clinically relevant postoperative complications within 30 days of surgery, excluding blood transfusion. The composite outcome comprised: (i) unplanned surgical intensive care unit (SICU) admission, (ii) requirement for postoperative ventilatory support, (iii) sepsis or shock, and (iv) surgical site infection (SSI).

SSIs were recorded based on postoperative clinical documentation during the 30-day follow-up period. Formal subclassification of SSI according to CDC criteria (superficial, deep, or organ-space) and grading by Clavien-Dindo severity were not systematically captured, and therefore SSI severity stratification was not performed. SSI was included in the composite outcome as a marker of postoperative morbidity consistent with prior observational studies evaluating intraoperative risk scores.

Blood transfusion was excluded from the composite endpoint to avoid incorporation bias, as EBL constitutes a component of the SAS. Outcome adjudication was performed by a single investigator blinded to the SAS category using prespecified clinical definitions. Complications were identified through inpatient medical records, including daily progress notes, intensive care unit records, operative and anesthesia documentation, laboratory results, and discharge summaries. Post-discharge events were captured through available hospital follow-up records. Formal inter-rater reliability assessment was not performed, as adjudication was conducted by a single trained investigator using standardized criteria.

Statistical analysis

All analyses were performed using Jamovi (version 2.6.44; The jamovi project (2025). Retrieved from https://www.jamovi.org). Continuous variables were summarized as mean ± standard deviation (SD) or median (interquartile range, IQR), as appropriate. Categorical variables were expressed as counts and percentages. Comparisons across SAS risk categories were performed using: (1) one-way analysis of variance (ANOVA) for normally distributed continuous variables, (2) Kruskal-Wallis test for non-normally distributed continuous variables, and (3) Chi-square test or Fisher’s exact test for categorical variables, as appropriate. 

Multivariable Analysis

Multivariable logistic regression was used to evaluate predictors of major postoperative complications, excluding blood transfusion. The primary model included four prespecified covariates: (1) SAS (ordinal, recoded so higher values indicate lower risk), (2) age, (3) duration of surgery, and (4) urgency of procedure (elective vs emergency). With approximately 40 outcome events, this model yielded an events-per-variable ratio of approximately 10, which is generally considered acceptable for logistic regression modeling.

A prespecified sensitivity analysis additionally included ASA physical status to assess the robustness of the association between the SAS and postoperative outcomes. Sensitivity analyses were interpreted cautiously, given the modest sample size and potential risk of overfitting. Odds ratios (ORs) with 95% confidence intervals (CIs) were estimated using Wald tests. Model fit was assessed using McFadden’s pseudo-R² and the Akaike Information Criterion (AIC) [11-12].

Handling of Missing Data

There were no missing values for any covariates included in the multivariable analyses. Logistic regression models were therefore conducted using complete-case analysis. One observation was excluded at the model level due to incomplete outcome data, resulting in an analytic sample size of 94 for regression analyses.

Model Performance

Discrimination was assessed using the area under the receiver operating characteristic (ROC) curve (AUC) with 95% CIs. An optimal cut-off was identified using Youden’s Index for exploratory purposes only. Diagnostic accuracy measures were reported descriptively. Given the modest sample size, the identified cut-off should be interpreted as data-driven and exploratory, and is not intended to define a clinical decision threshold without external validation.

Reporting standards

This manuscript adheres to the STROBE (Strengthening the Reporting of Observational Studies in Epidemiology) guidelines and TRIPOD (Transparent Reporting of a multivariable prediction model for Individual Prognosis or Diagnosis) principles were considered where applicable [13,14].

## Results

Baselines characteristics

A total of 95 patients were included in the study (mean age: 52.5 ± 18.7 years, range 18-85 years), of whom 48.4% were male. Most patients underwent general surgical procedures (57.9%), with the remainder undergoing orthopaedic surgeries (42.1%). Elective surgeries accounted for 55.8% of cases. The mean body weight was 63.1 ± 6.0 kg, and the median duration of surgery was 276 minutes (IQR: 236.5-306.0). Perioperative blood transfusion occurred in 42 of 95 patients (44.2%) and was analyzed separately from the composite outcome to avoid incorporation bias. 

Baseline associations with SAS

Baseline demographic and perioperative characteristics across SAS risk categories are summarized in Table 1. Elective surgery was more frequent among patients in the low-risk SAS category compared with those in the high-risk category. Higher ASA physical status and the presence of medical comorbidity were more common in patients classified as high risk by the SAS. Other baseline variables, including age, body weight, and duration of surgery, did not differ significantly across SAS risk categories.

**Table 1:** Baseline demographic, clinical, and perioperative characteristics of the study cohort stratified by Surgical Apgar Score risk categories Comparisons across Surgical Apgar Score risk categories (low, moderate, and high risk) were performed using one-way analysis of variance (ANOVA) for normally distributed continuous variables, Kruskal–Wallis test for non-normally distributed continuous variables, and Chi-square test or Fisher’s exact test (two-sided) for categorical variables, as appropriate. Test statistics are reported as F values for ANOVA, H values for the Kruskal–Wallis test, and χ² values for the Chi-square test, with corresponding degrees of freedom. A two-sided p-value <0.05 was considered statistically significant [11,12]. The Surgical Apgar Score was calculated based on estimated intraoperative blood loss, lowest heart rate, and lowest mean arterial pressure, as originally described by Gawande et al. [8]. ASA: American Society of Anesthesiologists physical status classification; SICU: surgical intensive care unit

Characteristic	Overall (N=95)	Low Risk (n=24)	Moderate Risk (n=47)	High Risk (n=24)	p-value	Statistical Test
Age (years), mean ± SD	52.5 ± 18.7	54.3 ± 18.5	53.0 ± 18.7	49.7 ± 19.5	0.590	One-way ANOVA
Male sex, n (%)	46 (48.4)	13 (54.2)	22 (46.8)	11 (45.8)	0.806	χ² test
Department, n (%)						
– General surgery	55 (57.9)	13 (54.2)	25 (53.2)	17 (70.8)		
– Orthopaedics	40 (42.1)	11 (45.8)	22 (46.8)	7 (29.2)		
Elective surgery, n (%)	53 (55.8)	19 (79.2)	27 (57.4)	7 (29.2)	0.002	χ² test
ASA physical status, n (%)					<0.001	Fisher’s exact
– ASA I–II	68 (71.6)	24 (100)	41 (87.2)	3 (12.5)		
– ASA III–IV	27 (28.4)	0 (0.0)	6 (12.8)	21 (87.5)		
Body weight (kg), mean ± SD	63.1 ± 6.0	64.1 ± 6.4	62.8 ± 6.2	62.5 ± 5.2	0.604	One-way ANOVA
Surgery duration (minutes), median (IQR)	276 (236.5–306.0)	283 (267.5–320.2)	264 (232.5–298.0)	272.5 (247.5–304.2)	0.306	Kruskal–Wallis
Medical comorbidity present, n (%)	17 (17.9)	1 (4.2)	5 (10.6)	11 (45.8)	<0.001	Fisher’s exact
Previous surgery, n (%)	12 (12.6)	2 (8.3)	3 (6.4)	7 (29.2)	0.018	χ² test

Multivariable logistic regression analysis

Multivariable logistic regression was performed to evaluate the association between the SAS and major postoperative complications, excluding blood transfusion. In the primary model adjusted for age, duration of surgery, and urgency of procedure (elective vs emergency), the SAS showed a trend toward association with major postoperative complications, although this did not reach statistical significance.

In a prespecified sensitivity analysis including ASA physical status, the SAS remained independently associated with major postoperative complications, excluding blood transfusion. The complication rate decreased after exclusion of blood transfusion to avoid incorporation bias, as transfusion is directly related to EBL, a component of the SAS. Each one-category improvement in the SAS risk category was associated with a reduction in the odds of major complications (adjusted OR ≈ 0.39; p = 0.035). ASA physical status was not independently associated with the outcome in this model. Results of the primary are presented in Table 2. 

**Table 2:** Multivariable logistic regression for major postoperative complications excluding blood transfusion (N = 95) Model: Adjusted for age, duration of surgery, and urgency of procedure (elective vs emergency) Odds ratios (ORs) and 95% confidence intervals (CIs) were estimated using multivariable logistic regression. The Surgical Apgar Score (SAS) was entered as an ordinal variable after recoding such that higher values indicate lower intraoperative risk. Major postoperative complications exclude blood transfusion. Odds ratios and p-values were derived from Wald tests in multivariable logistic regression [11,12]. *Higher SAS category values indicate lower SAS and higher intraoperative risk.

Predictor	Adjusted OR	95% CI	p-value
Surgical Apgar Score (per 1-category improvement)	0.57	0.31 – 1.07	0.088
Age (per year)	1	0.98 – 1.03	0.76
Surgery duration (per minute)	1.01	1.00 – 1.02	0.091
Elective surgery (vs emergency)	0.62	0.25 – 1.54	0.301

Model performance discrimination 

**Figure 1:**
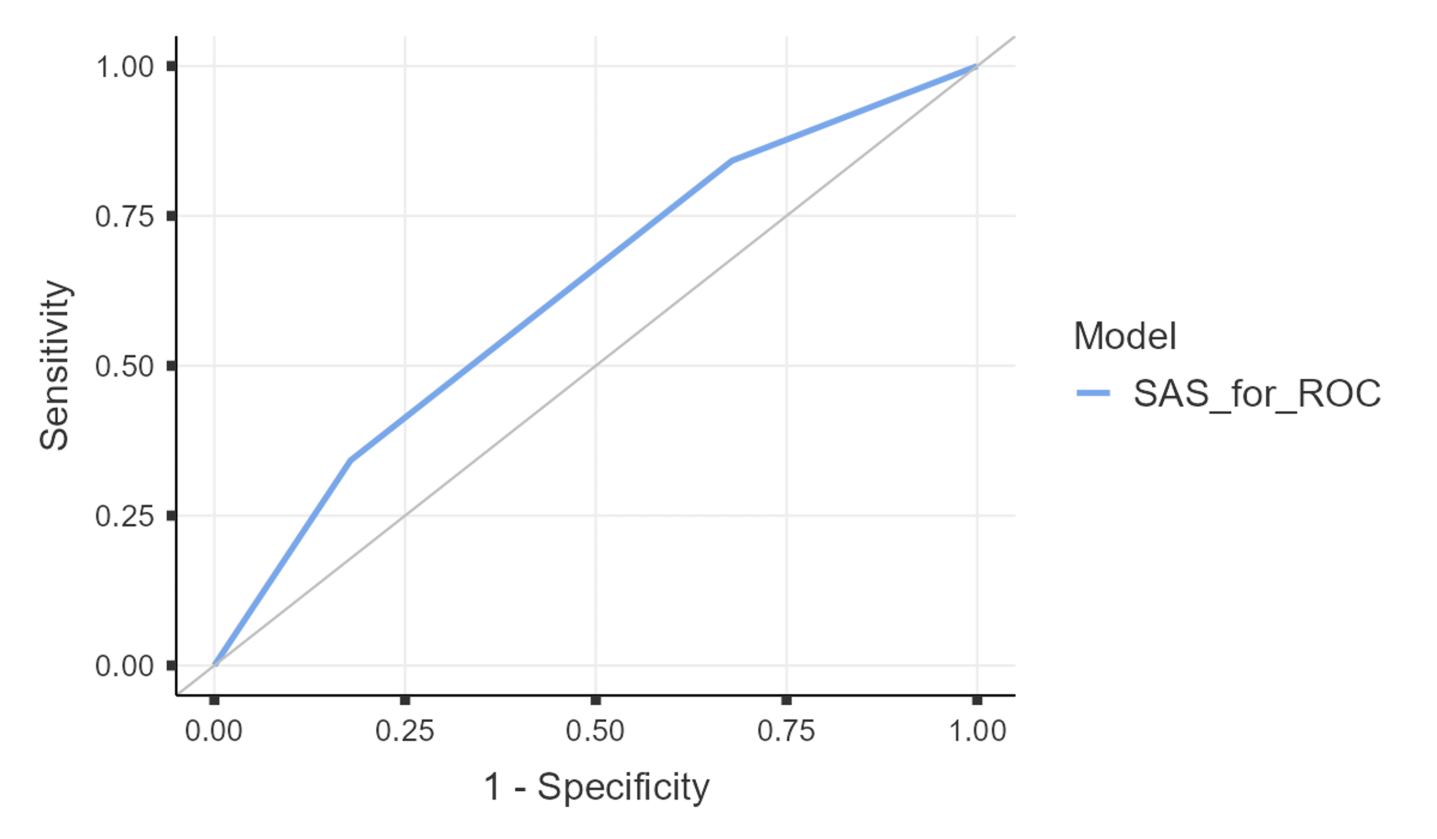
ROC curve illustrating the discriminative performance of the Surgical Apgar Score for predicting major postoperative complications excluding blood transfusion. The area under the curve (AUC) was 0.623 (95% CI: 0.517–0.728; p = 0.023), indicating limited to modest discrimination. The ROC curve is based on the Surgical Apgar Score and is presented for exploratory assessment of discrimination. The model is based on the Surgical Apgar Score as originally described by Gawande et al. [8]. ROC: receiver operating characteristic

ROC analysis demonstrated modest discriminative ability of the SAS for major postoperative complications (area under the curve (AUC) = 0.623; 95% CI 0.517-0.728; p = 0.023). At the optimal cut-off determined by Youden’s Index, sensitivity was 84.2%, and specificity was 32.1% (Figure 1), indicating a high true-positive rate but a substantial false-positive burden, which could translate into increased postoperative monitoring or resource utilization if applied as a standalone triage threshold.

Collinearity diagnostics were assessed for all predictors included in the multivariable analyses. Variance inflation factor (VIF) values were close to 1 for all covariates, indicating no evidence of problematic multicollinearity. Given the moderate sample size and correlation between preoperative and intraoperative risk measures, adjusted associations should be interpreted as exploratory. Diagnostic accuracy metrics across alternative thresholds are summarized descriptively in Table 3.

**Table 3:** Diagnostic accuracy measures for the Surgical Apgar Score in predicting major postoperative complications excluding blood transfusion at the optimal cut-off determined by Youden’s Index. Likelihood ratios are reported as unitless ratios. Diagnostic accuracy measures are descriptive and do not represent inferential testing.

Metric	Value (95% CI)
Sensitivity	84.2% (68.8–94.0)
Specificity	32.1% (20.3–46.0)
Positive Predictive Value	45.70%
Negative Predictive Value	75.00%
Positive Likelihood Ratio	1.24
Negative Likelihood Ratio	0.49
Accuracy	53.20%

## Discussion

In this prospective observational study, we evaluated the predictive performance of the SAS for major postoperative complications among adults undergoing major surgery in a tertiary care setting. Our findings demonstrate that while lower SAS values were generally associated with higher complication rates, the overall discriminative ability of the SAS was modest in this heterogeneous surgical cohort. ROC analysis showed an AUC of 0.623, indicating limited but statistically significant discrimination. At the optimal cut-off, the SAS demonstrated high sensitivity (84.2%) but low specificity (32.1%), suggesting that it may be more useful as a screening or triage tool rather than as a definitive standalone predictor of postoperative risk.

In the primary multivariable analysis adjusted for age, duration of surgery, and urgency of procedure, the SAS was not independently associated with major postoperative complications, excluding blood transfusion, although a nonsignificant trend toward lower odds of complications with improving SAS category was observed. However, in a prespecified sensitivity analysis that included ASA physical status, the SAS emerged as an independent predictor, with each one-category improvement in SAS being associated with a significant reduction in the odds of major postoperative complications. This finding suggests that the prognostic value of the SAS may be better appreciated when considered alongside established measures of preoperative physiological risk.

Our results are consistent with the 2023 systematic review by Mirzaiee et al., which synthesized evidence from 78 studies and concluded that the predictive accuracy of the SAS is highly context-dependent, with reported AUC values typically ranging between 0.6 and 0.7 depending on surgical specialty and patient population [10]. Their review emphasized that while the SAS is a practical and objective intraoperative tool, its optimal use is in conjunction with other clinical variables rather than as a standalone risk prediction model.

Several procedure-specific studies have reported stronger associations between the SAS and postoperative outcomes than those observed in our mixed cohort. For example, the study by Tracy et al. in emergency general surgery patients undergoing laparotomy demonstrated that lower SAS values independently predicted postoperative complications and mortality [15]. Similarly, Howbora et al. reported a strong independent association between low SAS and major complications following pancreaticoduodenectomy [16], Shakya et al. observed robust predictive performance of the SAS in neurosurgical populations [17], and Gudimetla et al. reported the usefulness of SAS in predicting the outcomes of hollow viscus perforation [18]. These findings suggest that the predictive strength of the SAS may be amplified in homogeneous, high-risk surgical populations, whereas its performance may be attenuated in more diverse, real-world cohorts such as ours.

Strengths and limitations

The modest discriminative performance observed in the present study underscores the limitations of using the SAS in isolation for individualized risk prediction across heterogeneous surgical populations. Nevertheless, the high sensitivity observed at the optimal cut-off indicates potential utility for early identification of patients who may benefit from closer postoperative monitoring or escalation of care. Consistent with prior recommendations, integrating the SAS with preoperative risk measures such as ASA physical status or other perioperative variables may improve overall risk stratification.

This study has several strengths, including its prospective design, systematic data collection, and evaluation of the SAS in a real-world mixed surgical population; however, important limitations should be acknowledged. First, the composite outcome included SSI without formal stratification by CDC depth (superficial vs deep/organ-space) or grading by Clavien-Dindo severity, as these data were not systematically captured a priori. Inclusion of lower-severity SSI may have increased the overall event rate and attenuated discrimination of the SAS for high-grade postoperative complications.

Second, detailed procedure-specific variables, including operative approach (open vs minimally invasive) and wound contamination class, were not systematically recorded. As the predictive performance of the SAS is known to be context-dependent, the absence of granular procedure-level classification limits direct comparability with procedure-specific validation studies and restricts interpretation to aggregate cohort-level performance. The SAS was available only as prespecified categories rather than as a continuous 0-10 score, precluding continuous modeling and reducing statistical power.

Third, the single-center design and moderate sample size limit statistical power and generalizability, and internal validation techniques such as bootstrapping were not performed to avoid overfitting. Accordingly, the reported discriminative performance should be interpreted as exploratory. Finally, blood transfusion was excluded from the composite endpoint to avoid incorporation bias, which may limit direct comparability with earlier studies that included transfusion as an outcome.

The SAS, thus, demonstrates modest but clinically meaningful predictive ability for major postoperative complications in heterogeneous surgical populations. While its standalone use for individual risk prediction appears limited, the SAS remains a valuable intraoperative tool for rapid risk assessment, particularly when integrated into multifactorial perioperative risk stratification strategies. Future large, multicenter prospective studies are warranted to further refine its role and to evaluate its performance within composite risk prediction models.

## Conclusions

In this prospective observational cohort study, the SAS demonstrated limited to modest discriminative ability for clinically relevant postoperative complications in a heterogeneous surgical population. The score was not independently associated with postoperative complications in the primary multivariable model; although an association was observed in sensitivity analysis after adjustment for ASA physical status, this finding should be interpreted cautiously given the modest sample size, potential conceptual overlap between preoperative and intraoperative risk measures, and the exploratory nature of the analysis.

Overall, these results suggest that the SAS has limited utility as a standalone risk stratification tool in heterogeneous surgical settings. At most, it may provide complementary intraoperative risk information when interpreted alongside established preoperative risk measures and clinical judgment. Further validation in larger, procedure-specific cohorts with standardized outcome definitions, calibration assessment, and internal validation is required before its role in perioperative risk assessment can be clearly defined.
